# Heat Balance Calculation and Energy Efficiency Analysis for Building Clusters Based on Psychrometric Chart

**DOI:** 10.3390/s21227606

**Published:** 2021-11-16

**Authors:** Shihai Yang, Huiling Su, Xun Dou, Mingming Chen, Yixuan Huang

**Affiliations:** 1 State Grid Jiangsu Electric Power Co., Ltd. Marketing Service Center, Nanjing 210019, China; ysh.young@163.com (S.Y.); rd1228@163.com (M.C.); huangyixuan_0304@163.com (Y.H.); 2 College of Energy and Electrical Engineering, Hohai University, Nanjing 210098, China; 3 State Grid Electric Power Demand Side Coordinated Control Technology Joint Laboratory, Nanjing 210019, China; 4 College of Electrical Engineering and Control Science, Nanjing TECH University, Nanjing 211816, China; dxnjut@njtech.edu.cn

**Keywords:** building clusters, heat balance, energy efficiency analysis, energy management, Psychrometric Chart, primary return air system, heating pipe network

## Abstract

How to perform accurate calculation of heat balance and quantitative analysis of energy efficiency for building clusters is an urgent problem to be solved to reduce building energy consumption and improve energy utilization efficiency. This article proposes a method for the heat balance calculation and energy efficiency analysis of building clusters based on enthalpy and humidity diagrams and applies it to the energy management of building clusters containing primary return air systems and heating pipe networks. Firstly, the basic structure and energy management principle of building clusters with a primary return air system and a heating pipe network were given, and the heat balance calculation and energy efficiency analysis method based on *i-d* diagram was proposed to realize the accurate calculation of heat load and the quantification of energy utilization. Secondly, the energy management model of the building cluster with a primary return air system and a heating pipe network was established to efficiently manage the indoor temperature and the heating schedule of ASHP, HN and HI. Finally, the proposed method was validated by calculation examples, and the results showed that the proposed method is beneficial for improving the energy economy and energy efficiency of building clusters.

## 1. Introduction

With the improvement of social living standards and the rising requirements of building comfort, building energy consumption has shown a continuous growth trend, bringing huge pressure to society, energy and the environment [1,2].

As the main body of the energy consumption of a building energy supply system, the air conditioning system accounted for about 33% of the total energy consumption of the building [3]. Existing studies had shown that the energy consumption of building energy supply systems could be reduced by about 20% to 30% through the optimal control of air conditioning systems without large-scale investment in renovation [4]. PRAS is the earliest, most basic and typical centralized air conditioning system to appear, and as the most basic form of air conditioning system, it is important to study the energy efficiency optimization of PARS-based building clusters [5]. The amount of heating (cooling) occupies most of the building energy consumption. Heat balance calculation, as one of the cores of air conditioning systems, was calculated by air conditioning to obtain the amount of heating (cooling) required to maintain the room temperature; therefore, optimizing control of air conditioning systems for energy management through accurate and efficient heat balance calculation methods, and thus quantifying and analyzing building energy efficiency, was the key to reducing building energy consumption.

In recent years, many advances have also been made in the heat balance calculation and energy efficiency analysis of building clusters. In terms of heat balance calculation, in [6], a prediction model for building energy consumption considering different heat production zones inside the building based on the building thermal storage characteristics was constructed. In [7], a building’s virtual energy storage system model was established based on building thermal inertia. An equivalent thermal parameter model for the central air conditioning system in public buildings was established in [8]. The RC model of the heat balance of the house was used to measure the heating load demand in [9]. The above study mainly focuses on the heat storage characteristics inside the building to establish an equivalent thermal parameter model for heat balance calculation. However, these heat balance calculation methods did not take into account the state parameters, such as enthalpy and humidity of indoor air, and only performed heat balance calculations with temperature as a thermal characteristic parameter.

In terms of building energy efficiency, an energy efficiency assessment index and its method were proposed based on time-series simulation, considering the specificity and time-series nature of commercial building loads in [10]. In [11], the Energy Advisor cloud energy efficiency consultant, to address the comprehensive energy consumption of buildings, was established. In [12], assessment metrics for evaluating electricity performance with respect to the building’s energy load were identified. In [13], the common application scenarios and technical routes of prediction models in the field of building energy efficiency optimization research were summarized, providing a comprehensive basis for researchers in building energy prediction. In [14], field tests of indoor heating environments in winter and summer were conducted for typical farm houses, and DeST-H energy simulation software was used in combination with orthogonal tests to propose the economical, energy-efficient and optimal experimental combination of building an energy efficiency improvement system suitable for farm houses.

In terms of energy management, a reinforcement learning-based energy management approach for building cluster end-use loads was established in [15]. In [16], an active distribution grid integrating smart buildings was established, considering wind power grid integration capabilities, an energy management strategy for smart buildings was proposed. In [17], three building cluster datasets were integrated for energy management and evaluation with respect to time, location, economic incentives and residents’ portfolios.

In terms of building energy saving, an energy prediction model for energy management in universities was developed and validated and an efficient system for multi-powered building energy was proposed in [18]. In [19], an operational mechanism for the whole process of property energy conservation management in commercial buildings was established, and three main energy conservation management approaches were proposed: behavioral energy conservation management, and the establishment of an internal energy conservation management platform and energy conservation renovation. In [20], energy demand estimates and retrofit scenarios for public types of buildings were constructed to derive the costs and benefits of energy efficiency measures for the public building stock. In [21], the building simulation models before and after the retrofit were modified in order to obtain the energy saving contribution of each retrofit technology of the building body by combining the actual retrofit effect of the building body.

The above studies mainly focused on building energy efficiency, energy consumption analysis, energy management and building energy saving. However, most of the existing building energy efficiency analysis is still regarded as a part of the assessment system, and the research on how to quantify energy efficiency accurately is not fine enough. The relationship between temperature and humidity was not clearly delineated. Little had been done on PRAS-based air conditioning principles for heat balance calculations and energy efficiency analysis of building clusters.

In the air-conditioning process, *i-d* diagram can determine parameters with multiple degrees of freedom of state change such as air temperature, moisture content, enthalpy, relative humidity [22], and it can accurately reflect the heat balance process in the air-conditioning process. Therefore, in this paper, based on the existing building energy modeling, a method for heat balance calculation and energy efficiency analysis of building clusters based on the *i-d* diagram was proposed and applied to the energy management of building clusters containing PRAS and heating pipe networks. It is of great significance to repair the limitation of building heat balance calculation, efficiency calculation and energy management research. The energy economy and energy efficiency of building clusters can also be improved. The innovation points of heat balance calculation and the energy efficiency analysis method proposed in this paper are as follows: the basic structure and energy management principle of building clusters, including PRAS and heating pipe network were constructed; heat balance calculation and energy efficiency analysis method based on the *i-d* diagram were proposed. The method was conducive to calculating the building heating load fine according to the indoor and outdoor air parameters and the set of indoor temperatures of the building cluster. A quantitative analysis of building energy efficiency was achieved by determining the standard coal equivalent conversion values of input and output energy within a building cluster. The energy management model of building clusters, including PRAS and a heating pipe network, was established. Considering external temperature, the average comfort of users and the energy price, based on heat balance calculation and energy efficiency analysis, the efficient management of indoor temperature and ASHP, HN and HI heating plan was realized.

## 2. The Basic Assumptions

### 2.1. The Basic Structure of a Building Cluster Containing PRAS and Heating Network

**Figure 1:**
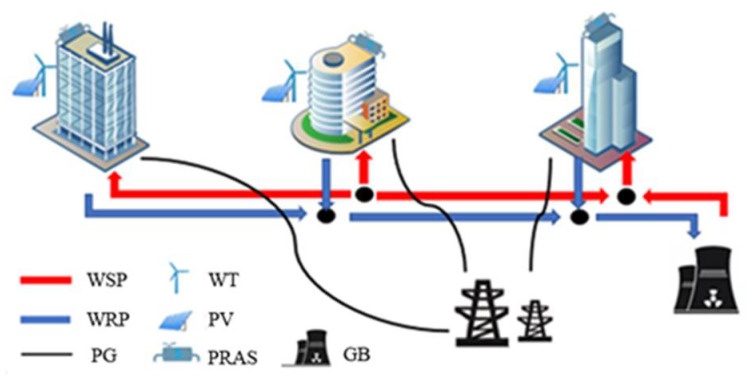
Basic structure of building cluster with primary return air system and heating pipe network.

In this paper, a building cluster with PRAS and a heating pipe network was constructed, and its basic structure was shown in Figure 1. The building cluster mainly contains a building unit (BU) and a heat supply pipe network. BU includes PRAS, wind turbine (WT) and photovoltaic (PV), and the heat supply pipe network includes the water supply pipe (WSP), the water return pipe (WRP) and the gas boiler (GB). The heating network includes a water supply pipe (WSP), a water return pipe (WRP), and a gas boiler (GB). Each BU purchases electricity from the power grid (PG) to meet the electrical load demand. Gas purchased from the natural gas network drives the GB for HN operation. Heat is produced through the HN and PRAS.

### 2.2. Building Cluster Energy Management Principle

Based on the basic structure of a building cluster containing a PRAS and a heating pipe network in Figure 1, the energy management principle of the building cluster was analyzed. Inside the BU, according to the PRAS working principle, the indoor temperature is managed and set based on the *i-d* diagram, and then the indoor heating quantity required by the heat balance was calculated. The indoor heat supply was provided by ASHP, HN and HI, respectively. ASHP needed to consume electrical energy, HN needed to be driven by GB, which consumes natural gas, and HI was caused by the time-delayed characteristics of the building indoor temperature, which was determined by the indoor temperature difference between adjacent periods. Therefore, in the energy management of building clusters, what needed to be managed externally as a whole was the amount of electricity and gas purchased. What needed to be managed for the internal BU was the indoor temperature setting and the heating power of ASHP and HN at each moment.

## 3. Heat Balance Calculation and Energy Efficiency Analysis Based on *i-d* Diagram

**Figure 2:**
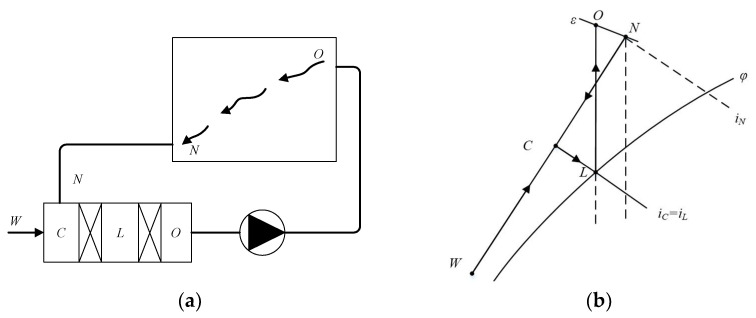
Heating principle of primary return air. (**a**) System schema; (**b**) Representation on the *i-d* diagrams.

The device of PRAS and the determination of the winter process on the *i-d* diagram are shown in Figure 2 [23].

**Figure 3:**
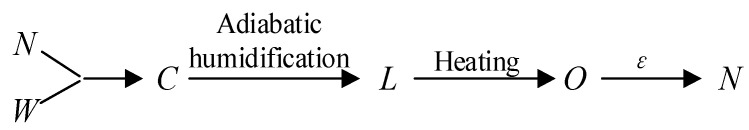
Winter process of primary return air system on *i-d* diagram.

Firstly, mark the outdoor state point *W* and indoor state point *N* on the *i-d* diagram, and make the indoor heat and humidity ratio line (*ε*) over the point *N*. According to the selected air supply volume *G*, calculate the air supply state point moisture content *do*, draw the *do* line, the intersection *O* of the line and *ε* is the air supply state point. In order to obtain the *O* point, the common method is to mix indoor and outdoor state *C* air by an adiabatic humidification process to the *L* point. The *L* point is called machine dew point, and it is generally located *φ* = 90% to 95% of the line, and heated from *L* to *O* point, then sent into the room and humidified into the indoor state *N*. Part of the indoor exhaust goes directly outdoors, and another part of the air conditioning room comes back to mix with the new air. Therefore, the whole treatment process is shown in Figure 3.

### 3.1. Heat Balance Calculations

Based on the heat treatment process of PRAS on the *i-d* diagram, the heat required for heat production *Q* can be obtained as:

(1) $$Q=G(i_{N}-i_{C})$$

(2) $$\frac{\bar{CN}}{\bar{WN}}=m,$$

where *m* is the fresh air ratio of the PRAS.

Then for the *k*th building in the building cluster the heat load *L*_h*,k,t*_ was calculated as in Equations (3) and (4).

(3) $$L_{h,k,t}=G[i_{\mathrm{in},k,t}(T_{\mathrm{in},k,t})-i_{C,k,t}]$$

(4) $$i_{C,k,t}=i_{\mathrm{in},k,t}T_{\mathrm{in},k,t}-m[i_{\mathrm{in},k,t}(T_{\mathrm{in},k,t})-i_{\mathrm{out},t}(T_{\mathrm{out},t})],$$

where *i*_in*,k,t*_(*T*_in*,k,t*_) denotes the enthalpy on the *i-d* diagram for the *k*th building in the building cluster at time *t* when the indoor temperature is set to *T*_in*,k,t*_. *i*_C*,k,t*_ is the enthalpy at point *C* of the PRAS heating process for the *k*th building in the building cluster at time *t*. *i*_out*,,t*_(*T*_out*,t*_) denotes the enthalpy on the *i-d* diagram for time *t* when the outdoor temperature is *T*_out*,t*_.

Equations (3) and (4) can be further simplified to obtain Equation (5), as shown below.

(5) $$L_{h,k,t}=Gm[i_{\mathrm{in},k,t}(T_{\mathrm{in},k,t})-i_{\mathrm{out},t}(T_{\mathrm{out},t})].$$

### 3.2. Energy Efficiency Analysis

On the basis of heat balance calculation to obtain the building heat load, the building energy efficiency calculation method proposed in this paper was as in Equations (6)–(15).

(6) $$\eta_{\mathrm{BEE}}=\frac{\sum_{i}\left(Q_{\mathrm{BEE},i}+S_{\mathrm{BEE},i,T}-S_{\mathrm{BEE},i,0}\right)}{\sum_{j}W_{\mathrm{BEE},j}+\sum_{m}W_{\mathrm{BEE},m}}\times 100\%$$

(7) $$Q_{\mathrm{BEE},i}=k_{e}Q_{e}+k_{h}Q_{h}$$

(8) $$W_{\mathrm{BEE},j}=k_{\mathrm{gas}}Q_{\mathrm{gas}}+k_{\mathrm{coal}}Q_{\mathrm{grid}}$$

(9) $$W_{\mathrm{BEE},m}=k_{e}(Q_{\mathrm{PV}}+Q_{\mathrm{WT}})$$

(10) $$Q_{e}=\sum_{k=1}^{3}\sum_{t=1}^{T}L_{e,k,t}\Delta t$$

(11) $$Q_{h}=\sum_{k=1}^{3}\sum_{t=1}^{T}L_{h,k,t}\Delta t$$

(12) $$Q_{\mathrm{gas}}=\sum_{k=1}^{3}\sum_{t=1}^{T}P_{G,k,t}\Delta t$$

(13) $$Q_{\mathrm{grid}}=\sum_{k=1}^{3}\sum_{t=1}^{T}P_{E,k,t}\Delta t$$

(14) $$Q_{\mathrm{PV}}=\sum_{k=1}^{3}\sum_{t=1}^{T}P_{\mathrm{PV},k,t}\Delta t$$

(15) $$Q_{\mathrm{WT}}=\sum_{k=1}^{3}\sum_{t=1}^{T}P_{\mathrm{WT},k,t}\Delta t,$$

where *η*_BEE_ denotes the building energy efficiency (combined energy efficiency) at the statistical time, %. *Q*_BEE,*I*_ denotes the standard coal equivalent converted value of the *i*th type of building energy output at the statistical time, kgce. *S*_BEE,*i*,*T*_ denotes the standard coal equivalent converted value of the terminal capacity of the *i*th type of energy output storage device at the statistical time, kgce.*S*_BEE,*i*,0_ denotes the standard coal equivalent converted value of the initial capacity of the *i*th type of energy output storage device at the statistical time, kgce. *W*_BEE,*j*_ denotes the standard coal equivalent converted value of the *j*th type of non-renewable energy input energy at the statistical time, kgce. *W*_BEE,*m*_ denotes the standard coal equivalent converted value of the energy converted from the *m*th type of renewable energy, such as wind and solar, into usable energy at the statistical time, kgce. *k*_e_, *k*_h_, *k*_gas_ and *k*_coal_ denote the discount factors for electricity, heat, natural gas, and raw coal, respectively. *Q*_e_ and *Q*_h_ denote the electricity and heat consumption of building clusters in the calculation period, respectively. *Q*_gas_, *Q*_grid_, *Q*_PV_ and *Q*_WT_ denote the gas purchases, electricity purchases from the grid, photovoltaic generation, and energy consumption in the calculation period, respectively. *P*_PV*,k,t*_ and *P*_WT*,k,t*_ are the photovoltaic power and wind power of the *k*th building in the building cluster. *P*_G,*k*,*t*_ is the gas purchase of the *k*th building in the building cluster at time *t*. *P*_E*,k,t*_ is the electricity purchase of the *k*th building in the building cluster at time *t*.

## 4. Building Cluster Energy Management Model Including PRAS and Heating Pipe Network

### 4.1. Objective Function

To minimize the operating cost of the building cluster, the objective function was established as shown in Equation (16).

(16) $$\min\;F=\sum_{k}f_{\mathrm{BU},k}=\sum_{t=1}^{T}\rho_{g}P_{G,k,t}+\rho_{e,t}\sum_{k}P_{E,k,t},$$

where *F* is the total operating cost of the building cluster, *f*_BU,*k*_ is the total operating cost of the *k*th building in the building cluster. *T* is the time period, *ρ*_g_ is the price of natural gas, *P*_G,*k*,*t*_ is the natural gas purchase amount of the *k*th building in the construction cluster period *t*, *ρ*_e,*t*_ is the electricity price in period *t*, *P*_E*,k,t*_ is the electricity purchase amount of the *k*th building in the construction cluster in period *t*.

### 4.2. Constraint Condition

1. Energy balance constraints
  (17) $$L_{e,k,t}+P_{A,k,t}=P_{G,k,t}+P_{\mathrm{PV},k,t}+P_{\mathrm{WT},k,t}$$
  (18) $$L_{h,k,t}=Q_{A,k,t}+Q_{\mathrm{hn},k,t}+Q_{\mathrm{hi},k,t},$$
  where *P*_A*,k,t,*_ *P*_G*,k,t,*_ *P*_PV*,k,t,*_ *P*_WT*,k,t,*_ is the electric power, purchase power, photovoltaic power and wind power of ASHP of the *k*th building in the building cluster, respectively.
2. Production model of air source heat pump [24]
  (19) $$Q_{A,k,t}=C_{\mathrm{OP},}{}_{t}P_{A,k,t}$$
  (20) $$P_{A,k,\min}\leq P_{A,k,t}\leq P_{A,k,\max},$$
  where *Q*_A*,k,t*_ is the heating power of the ASHP of the *k*th building in the building cluster. *C*_OP,*t*_ is the energy conversion efficiency COP value of the ASHP during the period *t*. *P*_A*,k,*max_ and *P*_A*,k,*min_ are the upper and lower limits of electrical power for ASHP of the *k*th building in the building cluster.
3. Gas turbine output model [25]
  (21) $$Q_{\mathrm{GB},t}=P_{G,t}H_{\mathrm{vg}}\eta_{\mathrm{GB}}$$
  (22) $$Q_{\mathrm{GB},\min}\leq Q_{\mathrm{GB},t}\leq Q_{\mathrm{GB},\max},$$
  where *Q*_GB,*t*_ is the thermal power of GB in the period *t*. *H*_vg_ is the thermal value of natural gas, and *η*_GB_ is the thermal production efficiency of GB. *Q*_GB,max_ and *Q*_GB,min_ are the upper and lower limits of GB thermal power respectively.
4. Heat net model

According to the literature [26,27], the radial heating network constraints include GB heating constraints, load acquisition constraints, node power fusion constraints, and pipe segment heat transfer constraints, specifically as follows:

(23) $$Q_{\mathrm{GB},t}=c\rho q_{\mathrm{GB},t}(T_{s,t}-T_{r,t})$$

(24) $$Q_{\mathrm{hn},k,t}=c\rho q_{hn,k,t}(T_{\mathrm{hs},k,t}-T_{\mathrm{hr},k,t})$$

(25) $$\sum_{j\in \Omega_{\mathrm{pipe}-}}T_{O,j,t}q_{j,t}=T_{I,j,t}\sum_{j\in \Omega_{\mathrm{pipe}+}}q_{j,t}$$

(26) $$(T_{b,jl,t}-T_{\mathrm{out},t}{)e}^{-\frac{L_{jl}}{Rc\rho q_{jl,t}}}=T_{e,jl,t}-T_{\mathrm{out},t},$$

where *c* is the specific heat capacity of hot water. *ρ* is the density of hot water. *q*_GB,*t*_ is the hot water flow at GB in the period *t. T*_s*,t*_ and *T*_r*,t*_ are the outlet water temperature and return water temperature at GB in the *t* period, respectively. *q*_hn,*k*,*t*_ is the hot water flow of the *k*th building in the period *t*. *T*_hs*,k,t*_ and *T*_hr*,k,t*_ are the effluent temperature and return water temperature of the *k*th building at period *t*, respectively. *Ω*_pipe-_ and *Ω*_pipe+_ are pipes with node *j* as the termination and starting node, respectively. *T*_O*,j,t*_ and *T*_I*,j,t*_ are the outlet water temperature and inlet water temperature of heat network pipe node *j* in the *t* period. *q_j,t_* is the hot water flow of the heat network pipeline at node *j* in the period *t*. *T*_b*,jl,t*_ and *T*_e*,jl,t*_ are the head temperature and end temperature of pipeline *jl* at the period *t*, respectively. *L_jl_* is the length of pipeline *jl*. R is the thermal resistance per unit length of the pipeline. *q_jl,t_* is the hot water flow in pipeline *jl* in the period *t*.

5. Thermal inertia model [7]
  The change of indoor heating balance is a slow process, which plays a certain buffer role in the process of air conditioning, and the specific expression is shown in Equation (27).
  (27) $$Q_{\mathrm{hi},k,t}=G(i_{\mathrm{in},k,t-1}-i_{\mathrm{in},k,t}),$$
  where *Q*_hi*,k,t*_ is the HI heating power of the *k*th building in the building cluster during period *t*.
6. Average comfort constraint
  (28) $$S_{k}=1-\frac{\sum_{t}\left|T_{\mathrm{em},k,t}-T_{\mathrm{in},k,t}\right|}{\sum_{t}T_{\mathrm{em},k,t}}$$
  (29) $$T_{\mathrm{in},k,min}\leq T_{\mathrm{in},k,t}\leq T_{\mathrm{in},k,max},$$
  where *S_k_* is the average comfort level of the $k_{th}$ building, and *T*_em*,k,t*_ is the most comfortable temperature of the $k_{th}$ building in period *t*. *T*_in*,k,*min_ and *T*_in*,k,*max_ are the upper and upper human comfort temperature of the $k_{th}$ building, respectively. In order to ensure the fairness of the heating effect of each building in the building cluster, the average satisfaction of all buildings is set to be equal, as follows:
  (30) $$S_{1}=S_{2}=\cdots =S_{k}.$$

### 4.3. Solution Flow

**Figure 4:**
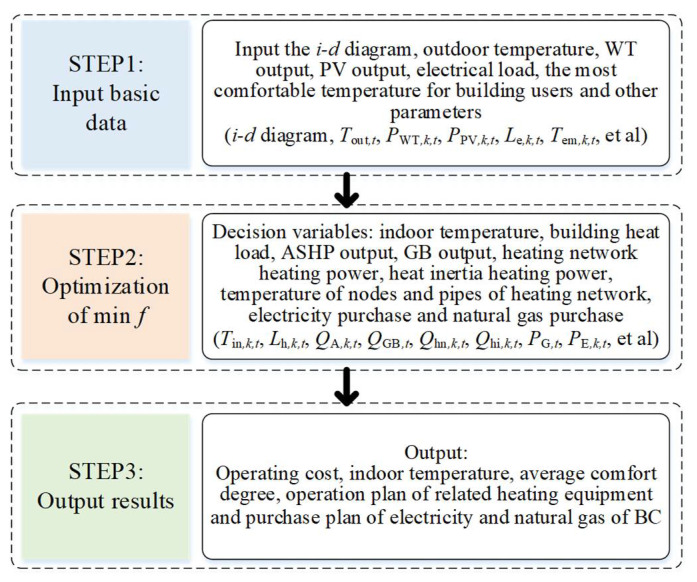
Solution flow.

The solution process of the model in this section was shown in Figure 4. First, input the *i-d* diagram, outdoor temperature, WT output, PV output, electrical load, the most comfortable temperature for building users, the mass flow of heating network pipelines and other basic parameters. Based on C#, the enthalpy-wet diagram calculator is compiled and embedded in the heat balance calculation model. Secondly, in view of the objective function and constraint conditions in Section 3.1 and Section 3.2, with the goal of minimizing the operating cost of the building cluster, the method of combining piecewise linearization and the sub-gradient method is used to linearize the discontinuous derivatives and nonlinear terms that exist in the constraints. Then, optimize decision variables such as indoor temperature, building heat load, ASHP output, GB output, HN heating power, HI heating power, heating network node and pipeline temperature, power purchase and gas purchase. Finally, output the operating cost, indoor temperature, average comfort, the operation plan of related heating equipment, and the purchase plan for the electricity and natural gas of building cluster.

## 5. Case Analysis

### 5.1. Basic Data

**Figure 5:**
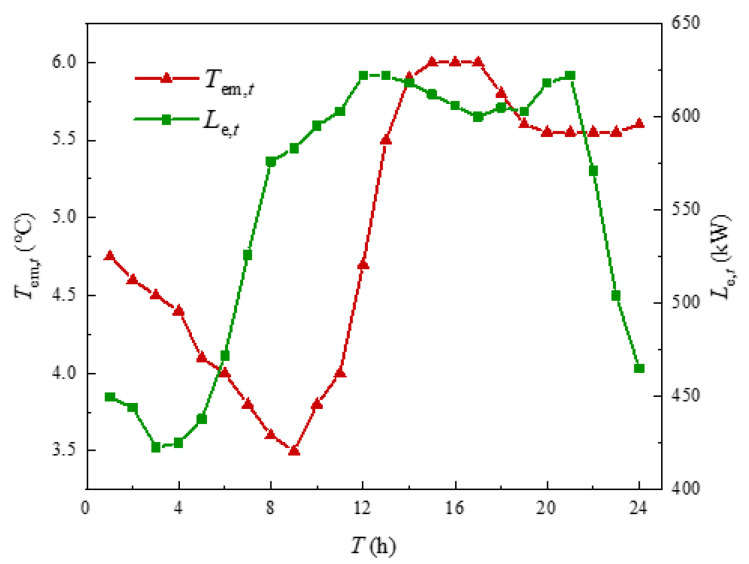
External temperature and electrical load.

**Figure 6:**
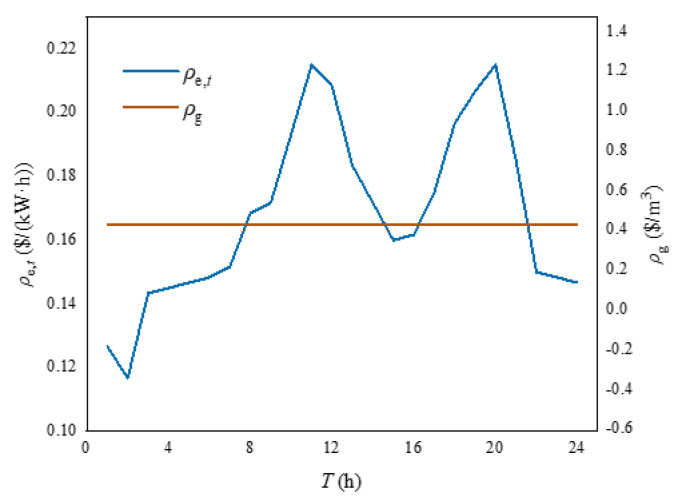
Electricity price and natural gas price.

**Figure 7:**
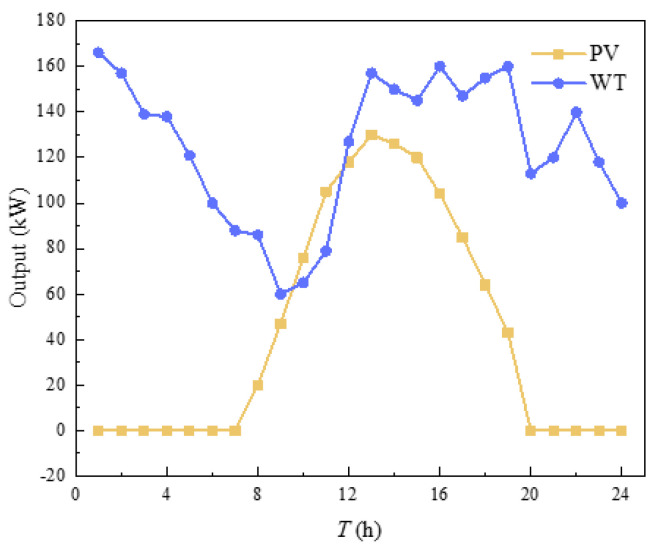
Output of PV and WT.

**Figure 8:**
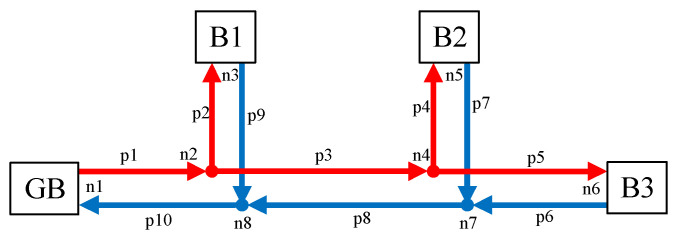
Node diagram of heating pipe network.

In order to verify the effectiveness of the heating balance calculation method proposed in this chapter, this paper was based on MATLAB and GAMS platform in the win10 operating system, i7CPU and 2.20 GHz processor environment for simulation and optimization analysis. The test case was constructed by combining the building cluster structure of Figure 1. The building cluster in this case contains three buildings. Because the physical processes of summer cooling and winter heating were quite different, this paper mainly solved the energy consumption problem of building cluster heat. Therefore, the calculation environment was set in winter. Assuming that one day in the local winter, the external temperature, electricity load curve, electricity price and natural gas price, WT output and PV output of the three buildings were all the same, as shown in Figure 5, Figure 6 and Figure 7 [28,29]. The capacities of GB and ASHP were 10 MW and 140 kW, respectively. The node diagram of the heating pipe network was shown in Figure 8. The parameters of the heating network pipes were shown in Table 1. The upper and lower limits of the water temperature were 85 °C and 60 °C. The remaining building cluster parameters were shown in Table 2 [30,31].

### 5.2. Result Validity Analysis

#### 5.2.1. Iteration Result

**Figure 9:**
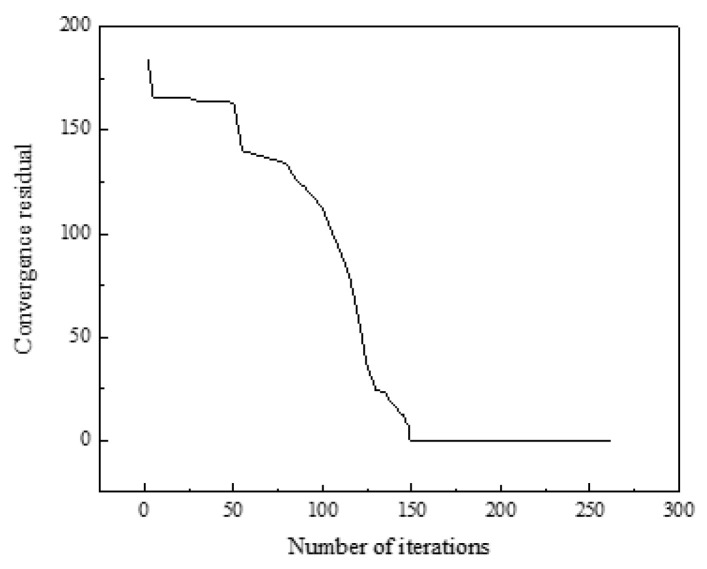
Iterative curve.

On the basis of the data in Section 5.1, the model was solved based on the solution process in Section 4.3. The iteration curve was shown in Figure 9, where the abscissa was the number of iterations and the ordinate was the convergence residual in the optimization process of the objective function. It can be seen that, after 253 iterations and the optimization results are obtained, the operating cost of the solved building cluster was 11,471.97$, and the average comfort level was 98%.

#### 5.2.2. Efficiency Analysis of Energy Management

In order to verify the effectiveness of the energy management method of building clusters with PRAS and heating pipe network based on the *i-d* diagram proposed in the article, two scenarios for comparative analysis were set up, as follows:

S1: Heat balance calculation and energy management of building clusters with PRAS and heating pipe network based on the *i-d* diagram;

S2: Heat balance calculation and energy management of building clusters with PRAS and heating pipe network without considering *i-d* diagram.

Where S1 was the method proposed in Section 4, and S2 was the energy management of the building cluster only for the set temperature of 23 °C without indoor air conditioning through the *i-d* diagram. The energy management costs of S1 and S2 are shown in Table 3.

According to Table 3, compared with S2, the total operating cost of S1 was reduced by 1.59%, which was more economical in terms of energy consumption. While the average comfort of S1 was reduced to 97.91% within the allowable range of user comfort. It can be seen that the heat balance calculation and energy management of building clusters with PRAS and heating network based on the *i-d* diagram were beneficial to reduce the operation cost of building clusters while ensuring the average comfort. However, the building energy efficiency of S2 was 0.3% higher than that of S1, mainly because the user comfort of S2 was 100%, the energy output on the numerator of the energy efficiency formula for S2 was higher than that for S1, the optimization goal was the lowest cost, and the natural gas energy input in denominator was increased, so the building energy efficiency of S2 was slightly improved compared with S1.

#### 5.2.3. Energy Management Scheme

**Figure 10:**
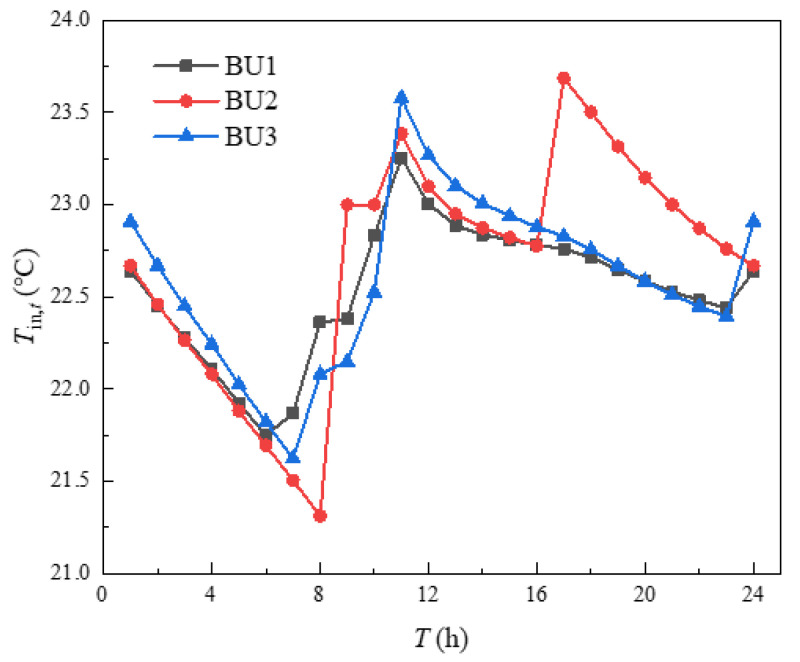
Indoor temperature management.

**Figure 11:**
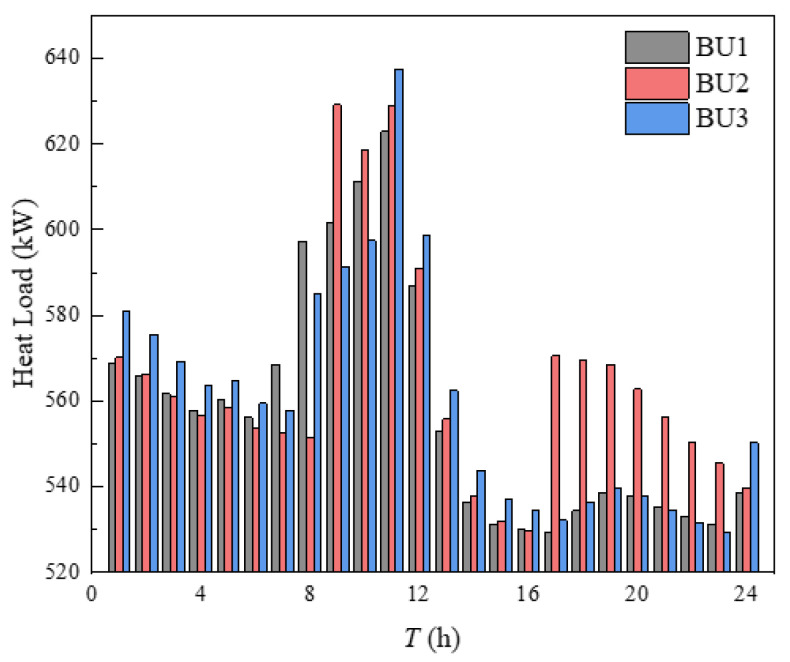
Heat load of building.

The indoor temperature management of three buildings in the building cluster was shown in Figure 10. The indoor heating load obtained by calculating the heat balance based on the *i-d* diagram was shown in Figure 11. It can be seen from Figure 10 that the indoor temperature settings of the three buildings fluctuate up and down around 23 °C, which was because the comfort of users and HI were utilized in energy management, and the indoor temperature settings were changed within the allowable range of temperature changes, so that the operation cost of building clusters in the whole period was minimized. The indoor temperature was set to decrease continuously at 1–8 h and 17–24 h, which was mainly due to the continuous decrease of outdoor temperature during these periods. If the indoor temperature was to be maintained at a high level, the heating load would be increased, and then the heating cost would be increased. The indoor temperature setting had a significant upward trend at 8–12 h. Combined with the gradually rising outdoor temperature, it can be seen that this appropriately increases the indoor temperature setting to store the heat load in advance. Over 12–16 h, the outdoor temperature changes little, while the indoor temperature setting decreases continuously, mainly because the HI provided by the higher temperature set at 12 h can be used for heating during these periods. In Figure 10, the temperature of BU3 at 24 h was obviously higher than that of BU1 and BU2, which was mainly because each BU needed to ensure the same temperature at the beginning and the end of the energy management cycle, which was convenient for periodic management, and the phenomenon in Figure 11 was the same. Finally, by comparing the outdoor temperature, indoor set temperature and indoor heat load, it can be concluded that, when the outdoor temperature was in the declining stage, the indoor temperature setting gradually decreases and the heat load decreases. When the outdoor temperature was in the rising stage, the indoor temperature setting gradually rises and the heat load increases. When the outdoor temperature was in a stable stage, the outdoor temperature setting gradually decreased and the heat load decreased.

**Figure 12:**
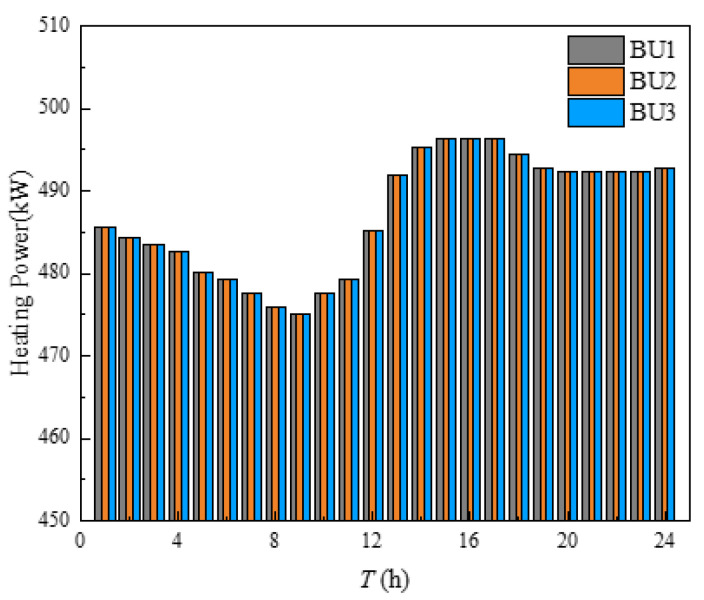
Heating power of GB.

The ASHP heat supply was shown in Figure 12. As can be seen in Figure 12, the ASHP heating output of three buildings was basically at full capacity. The ASHP output curves tend to coincide with the high and low external ambient temperature due to COP. It can be concluded that the heating cost of ASHP was lower than that of the heating network when the outside temperature was at 0–6 °C and the building clusters were preferentially heated by ASHP.

**Figure 13:**
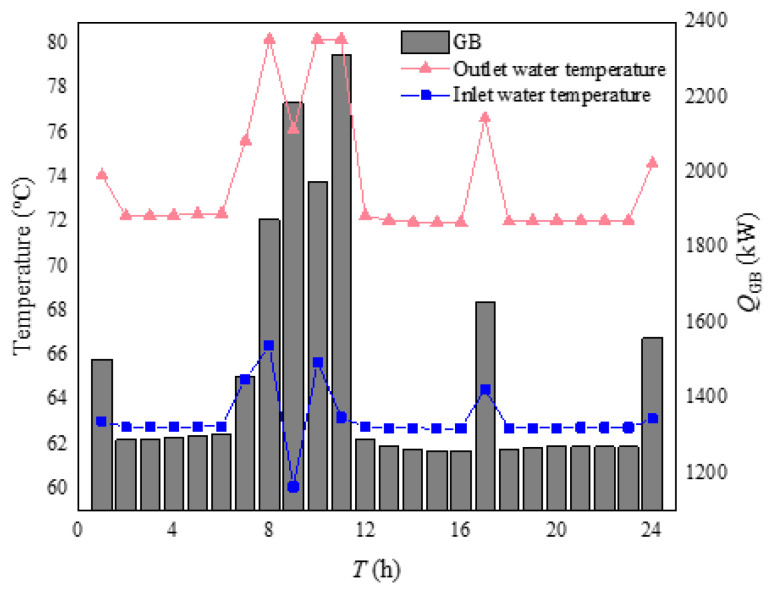
Heating power of GB and inlet and outlet water temperature.

**Figure 14:**
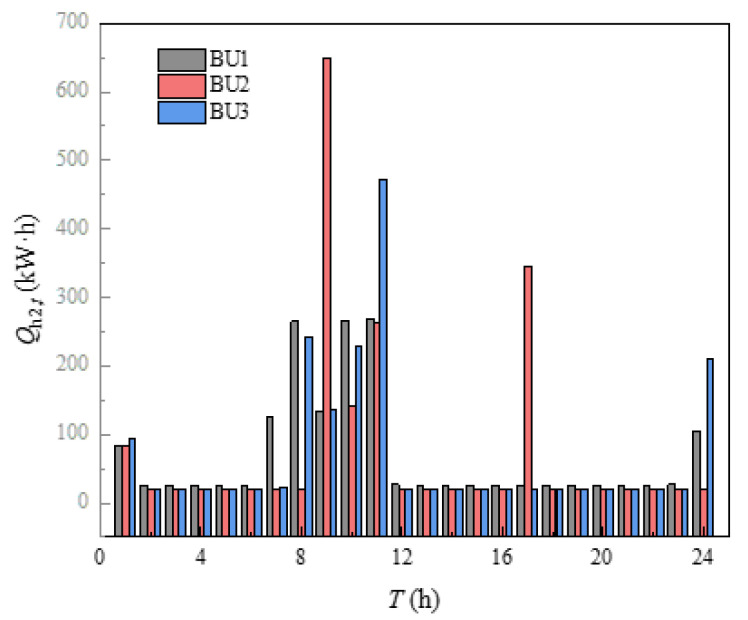
Heating output of heating pipe network.

The GB output and the heating pipe network supply were shown in Figure 13 and Figure 14. Firstly, the output level of GB was consistent with the heat output level of the heating network to the building. As can be seen from Figure 13, the higher the output of the electric boiler, the higher the output water temperature and the inlet water temperature. However, the core principle was that they had an increased temperature difference. Combining Figure 13 and Figure 14, there was a significant increase in the output of the GB and the heat supply network output from 7–11 h, mainly because the indoor temperature was lower during this period, the COP of the ASHP was smaller and the heating capacity was lower, so the heating needed to be supplied through the heating network, which also increases the output of the GB. It can be concluded that, during the 7–11 h period when the outdoor temperature was low, GB would increase its output to heat the indoor heat load to compensate for the decrease in heating capacity due to the decrease in the COP value of ASHP.

**Figure 15:**
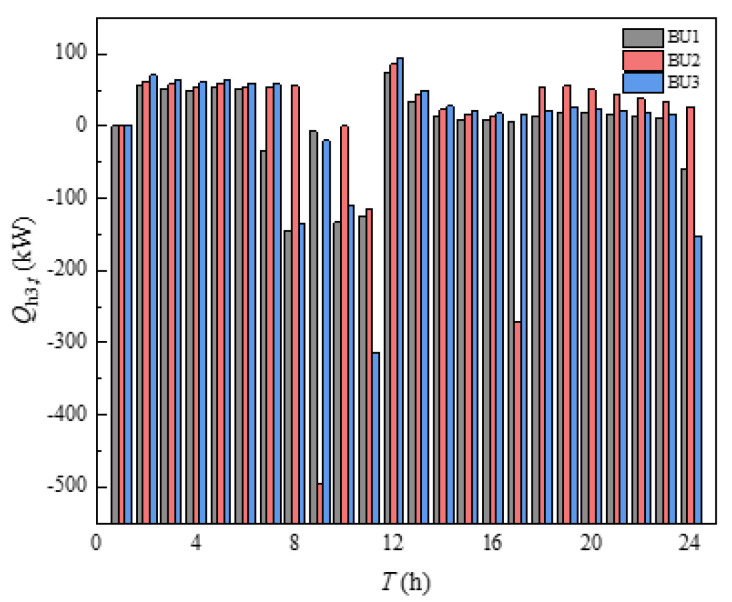
Heating power of thermal inertia.

The heating power of HI was shown in Figure 15, where a positive value indicates that the HI supplied the indoor heat load and a negative value indicated that the HI was compensated by the indoor heating. From Figure 15, it can be seen that the overall HI heating power was relatively smooth because the difference in indoor temperature settings was not very large. Several periods, such as 6–11 h and 24 h, when there was a significant drop in HI, were the periods when the indoor temperature settings were suddenly increased. The HI in other time periods was almost positive, indicating that the output power of HI was utilized to supply the indoor heat load. The HI of BU2 at 9 h and 17 h varied more compared with other BUs mainly because BU2 had a significant rise in temperature setting in these two moments, absorbing more heat provided by the heating network and storing heat in advance for the heat balance in subsequent time periods. It can be concluded that building clusters can use HI to supply indoor heat load, but some thermal compensation of HI was required for individual periods when the indoor temperature setting improved significantly.

### 5.3. Results Sensitivity Analysis

#### 5.3.1. Impact of Average Comfort on Energy Management of Building Clusters

**Figure 16:**
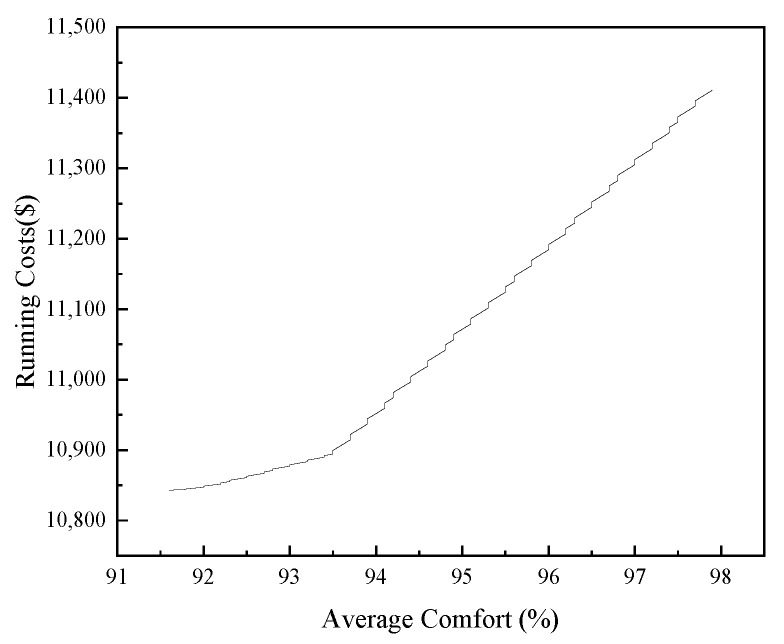
Relationship between average comfort and operating cost.

**Figure 17:**
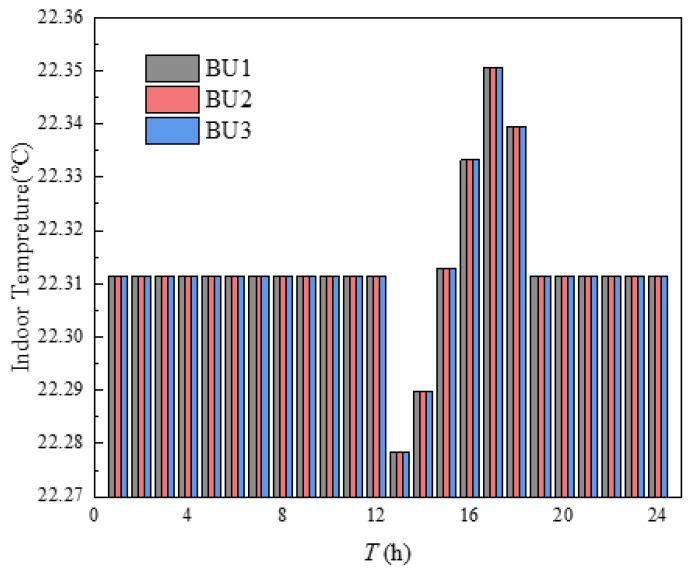
Indoor temperature setting with average comfort of 97%.

The influence of average comfort on energy management was analyzed by setting different lower limits of average comfort. After 100 different sets of average comfort settings, the relationship between average comfort and operating cost was obtained as shown in Figure 16. The influence of average comfort on energy management was analyzed by setting different lower limits of average comfort. After 100 different sets of average comfort settings, the relationship between average comfort and operating cost was obtained as shown in Figure 16. The slope of the curve in Figure 16 was defined as the marginal cost of the average comfort level on the energy management of the building cluster. From Figure 17, it can be seen that, as the average comfort level increases, the operating cost of the building cluster also rises gradually. When the average comfort level was between 93.6% and 98%, the marginal cost of the average comfort level was roughly 109.30, and the total operating cost of the building cluster rises rapidly. When the average comfort level was between 92% and 93.6%, the marginal cost was roughly 32.60, and the total operating cost of the building cluster rises less rapidly. When the average comfort level was between 91.6% and 92%, the marginal cost was roughly 11.36, and the rate of increase of the total operating cost of the building cluster decreases again. It can be seen that the average comfort level has the greatest impact on the total running cost of the building cluster when the average comfort level was between 93.6% and 98%. However, as the average comfort level decreased, the impact gradually decreased and the running cost of the building cluster decreased gradually and slowly.

**Figure 18:**
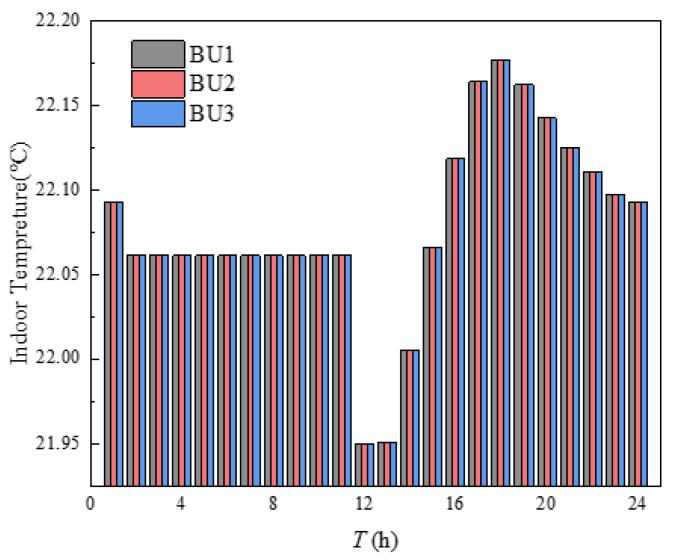
Indoor temperature setting with average comfort of 96%.

The points in Figure 16 with an average comfort level of 97% and 96% were taken to analyze the variability of indoor temperature settings at different average comfort levels, as shown in Figure 17 and Figure 18, respectively. According to Figure 17 and Figure 18, the indoor temperature settings of the three buildings became consistent and the points in time when the temperatures were equal became more frequent, which was mainly caused when the average satisfaction of all buildings was set equal in order to ensure the fairness of the heating effect of each building in the building cluster. Both had a low to high indoor temperature setting at 13–16 h, mainly due to the higher constant temperature in the previous periods. The outdoor temperature was high at this time of the year, allowing the HI to be used for continuous heating, and the indoor temperature setting was gradually increased as the thermal power that the HI can provide continued to decrease over time. Comparing Figure 10, Figure 17 and Figure 18, the mean values of the indoor temperature settings were 22.61 °C, 22.31 °C and 22.08 °C, respectively. The main reason was that an overall reduction in indoor temperature reduces the heat load, which in turn reduced the operating costs of the building cluster. It can be seen that, as the average comfort level decreases, there is an overall decrease in the indoor temperature setting. However, the role of HI in this process was mainly reflected in 13–16 h by the setting of the room temperature from low to high.

#### 5.3.2. Impact of Heat Use in Building Clusters on the Node and Pipe Temperatures of the Heating Network

**Figure 19:**
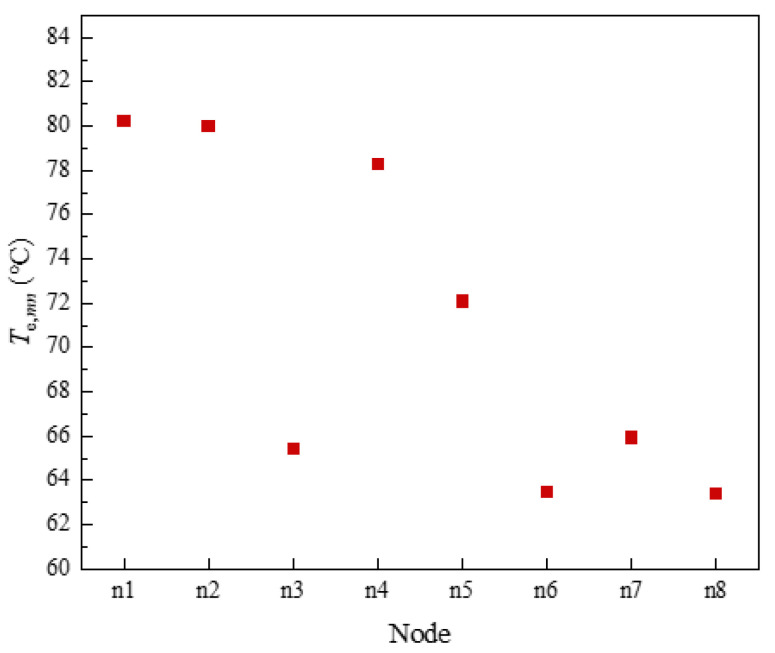
Node temperature of heating pipe network.

The temperature variation at each node of the heating pipe network was shown in Figure 19. According to Figure 19, it can be seen that node 1, node 2 and node 4, as GB outlet nodes and water supply pipeline nodes, had higher temperatures, which were above 70 °C. The temperature of the GB outlet node was the highest, and the temperature of the water supply pipeline node gradually decreased with the direction of pipeline flow. Node 3, node 5, node 6, node 7 and node 8, as load nodes and return water piping nodes, were at a lower temperature, below 68 degrees Celsius. The load node temperature was lower than the water piping node temperature. The temperature of node 7 was higher than that of node 6 because node 7, as a return water piping node, incorporates the temperature of the return water flowing from nodes 5 and 6 to node 7. The higher temperature of node 6 caused the temperature of fused node 7 to be higher than that of node 6. It can be seen that the temperature of the water supply pipe node and the return pipe node in the heating pipe network gradually decreased with the flow of the pipe. The temperature of the GB outlet node and the water supply piping node was higher than the temperature of the load node and the return piping node. The temperature of the return piping node and the temperature of the load node should be determined as high or low according to the actual flow rate.

**Figure 20:**
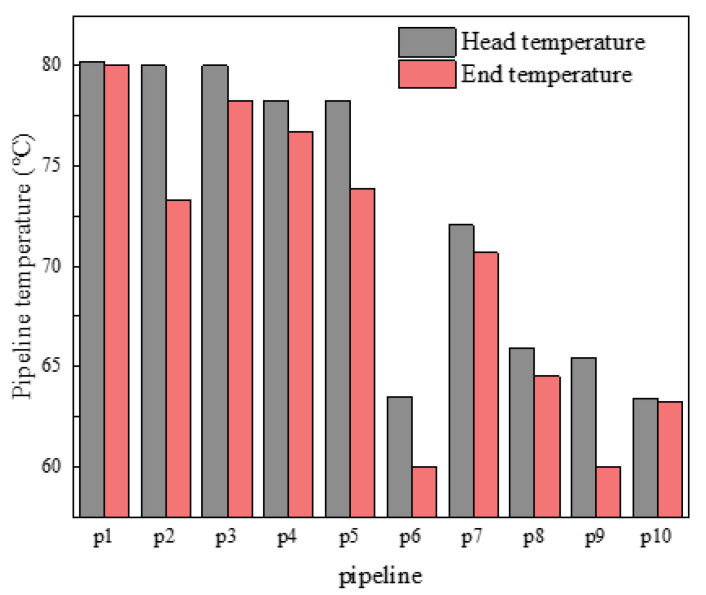
Temperature at the head and end of the pipe.

At 11 h, the temperature of the first end of the heating pipe was shown in Figure 20. It can be seen from Figure 20 that there was a large difference in temperature loss between the first and last ends of the pipe. Pipe 2 had the largest loss of 6.70 °C and pipe 10 had the smallest loss of 0.17 °C. According to Equation (16), it was known that the heat network loss was closely related to the length of the pipe, the external temperature and the flow of water. In Figure 20, the difference in temperature loss was mainly due to the difference in length and flow rate of different pipes. Based on the parameters of pipe No. 2 and No. 10 in Table 1, it was not difficult to conclude the reason for such a gap in temperature loss. Therefore, a reasonable design of pipe length and an improvement of water flow can effectively control the temperature loss of the heating pipe network.

## 6. Conclusions

In this paper, a heat balance calculation and energy efficiency analysis method based on the *i-d* diagram for building clusters was proposed and applied to indoor temperature and energy supply and the consumption plan management of building clusters. The following conclusions were mainly obtained through the calculation examples in this paper.

(1) The method proposed in this paper was capable of refining the calculation of building heating loads, quantitatively analyzing building energy efficiency, taking into account the average comfort level of building clusters, efficiently managing indoor temperatures and the heating schedules of ASHP, HN, and HI, improving operational economy and reducing energy consumption by considering many factors such as the enthalpy and humidity of indoor and outdoor air in building clusters and the set indoor temperature;
(2) Using the method of this paper, the impact on the total operating cost of the building cluster was greatest when the average comfort level was between 93.6% and 98% under the environment of 3–6 °C outside temperature in winter, and the marginal cost of the average comfort level reaches 109.30, and the operating cost could be effectively reduced by reducing the average comfort level. As the average comfort level decreases, the impact gradually decreases and the marginal cost decreased to 11.36 when the average comfort level decreased to 91.6–92%, and it was not recommended to reduce the average comfort level again in order to reduce the operating cost.

The energy system input part of this paper did not involve energy storage technology. In the subsequent research, this paper combined the energy storage system to realize the new energy distribution and storage business, and comprehensively distribute each energy source to achieve economic cost minimization and energy efficiency optimization under the premise of ensuring comfort.

## Figures and Tables

**Table 1:** Parameters of heating pipe network.

Pipe Number	Length (m)	Flow (m^3^/h)
p1	100	120
p2	800	30
p3	600	90
p4	300	50
p5	700	40
p6	700	40
p7	300	50
p8	600	90
p9	800	30
p10	100	120

**Table 2:** Parameters of building cluster.

Parameter Name	Parameter Value
*G*	150 kg/h
*m*	15%
*H*_vg_	9.88 kJ/m^3^
*η*_GB_	0.9
*R*	265 (m·°C)/kW
*c*	4.2 kJ/(kg·°C)
*ρ*	934.67 kg/m^3^

**Table 3:** Comparison of building cluster energy management results in different scenarios.

Result	S1	S3
*F* ($)	11,480.48	11,666.45
*S_k_* (%)	97.91	100
*η*_BEE_ (%)	22.30	22.60
*F* ($)	11,480.48	11,666.45

## Data Availability

Not applicable.

## References

[1] The International Energy Agency: The World Energy Brief Statistics in 2012. http://www.trqgy.cn/report/201303/23344.html.

[2] Wang X., Kendrick C., Ogden R., Walliman N., Baiche B. (2013). A case study on energy consumption and overheating for a UK industrial building with roof lights. Appl. Energy.

[3] US DOE (2009). Buildings Energy Data Book[EB/OL]. http://buildingsdatabook.eren.doe.gov.

[4] Marc G., Tobias M., Thomas H. (2018). Estimation of the energy demand of electric buses based on real-world data for large-scale public transport networks. Appl. Energy.

[5] Jing L. (2010). Energy saving comparing between primary return air system with secondary return air system in dehumidification. Refrig. Air Cond..

[6] Chen H., Li Z., Jiang T., Li X., Zhang R.F., Li G.Q. (2019). A Flexible Control Strategy for Intelligent Building Energy Use Based on Model Predictive Control. Autom. Power Syst..

[7] Jin X., Mu Y., Jia H., Yu X., Chen N. (2017). Optimal scheduling method for a combined cooling, heating and power building microgrid considering virtual storage system at demand side. Proc. CSEE.

[8] Xu Q.S., Yang C.X., Yan Q.G. (2016). Daily peak cutting strategy of power load for large-scale air conditioning. Grid Technol..

[9] Baeten B., Rogiers F., Helsen L. (2017). Reduction of heat pump induced peak electricity use and required generation capacity through thermal energy storage and demand response. Appl. Energy.

[10] Li H.B., Zhao Y.M., Liu G.W., Jiang S.Y., Zhao Z.J. (2020). Energy Efficiency of Commercial Building AC and DC Distribution System Based on Time iming Simulation. Electrotech. J..

[11] Xing L. (2019). Schneider Electric: Using the Energy Advisor Cloud Energy Efficiency Consultant as the accelerator to release the greater potential for building energy efficiency. Electr. Technol..

[12] Shi Y., Shen J., Zhang C. (2021). Study on Cluster Building Coordination Control Scheme Based on Load Energy Efficiency Assessment. Electr. Age.

[13] Zhu M.Y., Pan Y.Q., Lv Y., Wang Q.J., Li Y.M., Huang Z.Z., Tao Q.B. (2020). Application Review of Energy Consumption Prediction Models in Building Energy Efficiency Optimization. Build. Sci..

[14] Wang N.Y., Zhao J.Y., Wang Q. (2021). Building Energy Efficiency System of Rural Residential Buildings in Guanzhong Area, China. Build. Energy Effic..

[15] Ge S., Li J., Liu H., He X. (2021). Demand-Side Energy Management Method for Building Clusters Applying Reinforcement Learning. Electr. Power Constr..

[16] Li Z.N., Su S., Jin X.L., Chen H.H., Wei C.H., Zhao Z.M. (2021). Energy Management Strategy of Smart Buildings for Improving Wind Power Accommodation Ability [J/OL]. Power Syst. Technol..

[17] Hu Q., Fang X., Li F., Xu X., Chen C.F., Hu H. An approach to assess the responsive residential demand to financial incentives. Proceedings of the 2015 IEEE Power & Energy Society General Meeting.

[18] Yoon S.H., Kim S.Y., Park G.H., Kim Y.K., Cho C.H., Park B.H. (2018). Multiple power-based building energy management system for efficient management of building energy*-*ScienceDirect. Sustain. Cities Soc..

[19] Deng X. (2016). Energy saving management mode of commercial buildings based on property management. Shenzhen Daxue Xuebao (Ligong Ban)/J. Shenzhen Univ. Ence Eng..

[20] Novikova A., Szalay Z., Horváth M., Becker J., Simaku G., Csoknyai T. (2020). Assessment of energy-saving potential, associated costs and co-benefits of public buildings in Albania. Energy Effic..

[21] Liu X., Wang C.C., Feng G.H., Yin Z.K., Li Z.H. (2020). Contribution Rate of Energy-saving Renovation of Existing Non-energy-energy-saving Residential Buildings in Typical Cities in Severe Cold Regions. Build. Sci..

[22] Erdélyi P., Rajkó R. (2016). Using Interactive Psychrometric Charts to Visualize and Explore Psychrometric Processes. J. Chem. Educ..

[23] Zhao R. (1994). Air Conditioning-Version 3.

[24] Song M., Deng S., Dang C., Mao N., Wang Z. (2018). Review on improvement for air source heat pump units during frosting and defrosting. Appl. Energy.

[25] Ren S., Dou X., Wang Z., Wang J., Wang X. (2020). Wang Medium- and Long-Term Integrated Demand Response of Integrated Energy System Based on System Dynamics. Energies.

[26] Li Z., Wu W., Shahidehpour M., Wang J., Zhang B. Combined heat and power dispatch considering pipeline energy storage of district heating network. Proceedings of the 2017 IEEE Power & Energy Society General Meeting.

[27] Li Z., Wu W., Wang J., Zhang B., Zheng T. (2016). Transmission-Constrained Unit Commitment Considering Combined Electricity and District Heating Networks. IEEE Trans. Sustain. Energy.

[28] DIW Berlin: DIETER. https://www.diw.de/de/diw_01.c.508843.de/forschung_beratung/nachhaltigkeit/umwelt/verkehr/energie/modelle/dieter/dieter.html.

[29] Chirambo D. (2016). Addressing the renewable energy financing gap in Africa to promote universal energy access: Integrated renewable energy financing in Malawi. Renew. Sustain. Energy Rev..

[30] Dou X., Wang J., Wang Z., Ding T., Wang S.Z. (2021). A decentralized multi-energy resources aggregation strategy based on bi-level interactive transactions of virtual energy plant. Int. J. Elect. Power Energy Syst..

[31] Dou X., Wang J., Wang Z., Li L.J., Bai L.Q., Ren S.H. (2020). A dynamic time-interval model predictive control based dispatch method for integrated energy system. J. Mod. Power Syst. Clean Energy.

